# Interest in and Barriers to Telehealth Uptake in an Obstetric and Pediatric Medicaid Population

**DOI:** 10.7759/cureus.30148

**Published:** 2022-10-10

**Authors:** Mallory Novack, Kristina Roloff, Guillermo J Valenzuela

**Affiliations:** 1 Obstetrics and Gynecology, Arrowhead Regional Medical Center, Colton, USA

**Keywords:** medicaid barriers to telehealth, telehealth in obstetrics, barriers, medicaid population, telehealth

## Abstract

Telehealth has been shown to be generally well accepted by patients and physicians with an increasing desire and utilization of this practice since the COVID-19 pandemic. However, studies looking specifically at the United States’ low socioeconomic populations’ interest in and barriers to accessing Telehealth care are limited. In this study, we performed a survey to determine the interest of pediatric and obstetric patients on and the reasons they may or may not choose Telehealth visits in a practice that serves solely California Medicaid (Medi-Cal) patients. A total of 636 patients completed the questionnaire, 383 (60%) from an obstetric practice and 253 (40%) from a pediatric practice. The majority expressed that they were not interested in Telehealth (N=407, 64%), and 228 (36%) stated interest. Interest in Telehealth was related to domains of cost, access, and attitude (P<0.005 for each) for the entire sample. The highest scores (preference toward Telehealth) were noted in the domain of enjoyment; this suggests that both pediatric and obstetric patients may decline Telehealth in favor of in-person meetings simply because they like meeting with their provider. Despite readily available technology/access for Telehealth visits in low socioeconomic patients, in-person healthcare may be preferred by this patient population. In the world of changing healthcare delivery and epidemics, in-person visits are an important option for disadvantaged patients.

## Introduction

Telehealth is not a novel practice but has gained interest in recent years, especially since the COVID-19 pandemic has spread worldwide. Financial, regulatory, and technological challenges in the past have made it difficult to advance the wide implementation of Telehealth. “Evidence suggests that Telehealth provides comparable health outcomes when compared with traditional methods of healthcare delivery without compromising the patient-physician relationship [1,2].” Telehealth also has been shown to have a positive patient experience and can “enhance patient satisfaction and improve patient engagement” [1]. Patients have identified Telehealth as being more convenient and effective, providing easier communication, and having enhanced privacy and comfort from their home domain [3]. Lastly, Telehealth has been shown to be generally well accepted by patients and physicians with an increasing desire and utilization of this practice since the COVID-19 pandemic [4].

However, studies looking specifically at Telehealth care in low socioeconomic patients are limited. Despite advancing technology and its availability, financial disparities in smartphone access and internet services may contribute to barriers to Telehealth advancement in this low socioeconomic patient population [5]. For household incomes under the poverty line of $30,000 annual income, 29% do not have smartphones, 44% do not have access to broadband internet, and 46% do not have access to computers [5].

There have been reports of underutilization of Telehealth services in vulnerable populations during the COVID-19 pandemic, which may even exacerbate current healthcare disparities [6-9]. The reasons for the different uptake of Telehealth services based on socioeconomic, racial, and demographic factors are largely understudied but deserve attention [10,11]. We know of potential limitations in technology, but few studies have determined interest in and specific barriers to Telehealth utilization in disadvantaged patients.

In this study, we analyzed a survey that was previously collected as part of a practice management project to determine Telehealth interests in a California Medicaid (Medi-Cal) pediatric and obstetric patient population. We also aimed to gain an understanding of the barriers surrounding Telehealth in low socioeconomic populations and better understand strategies to make Telehealth a viable adjunct to office visits.

This article was previously presented at a local hospital research day, the annual Arrowhead Regional Medical Center’s Resident/Fellow Research Day on May 27, 2022.

## Materials and methods

This is a retrospective study of obstetric and pediatric patients’ parents/guardians’ reasons for choosing in-office or Telehealth care. Patients or their parents/guardians completed a questionnaire about Telehealth during a routine office visit. This survey was administered as part of a practice management project (to determine if there was interest in Telehealth care) at a scheduled outpatient visit during a four-week study time frame between March 31, 2021, and May 6, 2021. Patients were asked about their preferences in the following domains related to Telehealth: cost, access, and attitude. Details of how visits would be conducted were not disclosed in the survey.

Patients were asked if they were interested in Telehealth (yes or no) and, if so, what kind of device they would use (iPhone, Android, computer, or other) and if they would prefer text or email communication, two-way audiovisual visits, or audio (telephone)-only visits. They were also queried in domains of cost, access, and attitude through agreeing or disagreeing with statements related to Telehealth. The survey answers were constructed using a 5-point Likert scale from strongly disagree (1) to strongly agree (5) with survey statements (Appendix).

Results were ordered so that higher scores would suggest interest, ability, or desire for Telehealth, and a lower score would favor in-person care. The internal reliability of the scale was calculated using Cronbach’s alpha. Cost was constructed using a mean of questions about equipment, internet service, and data charges, and access was calculated as the mean of questions about the timeliness of care and transportation. Attitude was assessed as the mean of questions about the complexity of health problems, confidence in providers, and enjoyment of in-person visits (Table 1).

**Table 1:** Survey domains and statements. Respondents were queried on a 5-point Likert scale from strongly agree to strongly disagree with these statements.

Domain	Survey statement	Cronbach’s alpha
Cost	I have the equipment (smartphone or computer) needed for telehealth (equipment).	0.497
Cost	I have internet access that can support televideo visits (internet).	0.499
Cost	I am worried about the costs of telehealth services such as text or data charges (data charges).	0.626
Access	Telehealth will help me see my provider timelier or more often (timeliness).	0.477
Access	I have a hard time making it to my visits due to transportation problems (transportation).	0.536
Attitude	My health conditions are too complicated for telehealth (complexity of care).	0.640
Attitude	I am confident that my provider will be able to care for me through telehealth (confidence in provider).	0.570
Attitude	I enjoy meeting with my provider in person (enjoyment).	0.741

Survey results were tabulated, and descriptive statistics were calculated. Comparisons by practice type (obstetric or pediatrics) for Likert scale findings across the domains outlined above were performed by t-testing the mean. Similarly, comparisons by those who expressed interest in Telehealth with those who did not were conducted for all patients and for obstetric and pediatric patients by t-test of Likert scale means. Binomial logistic regression was used to model the relative contributions of each domain to interest in Telehealth.

This study was determined to be exempt from Institutional Review Board (IRB) review by Sterling IRB as the survey was previously collected as a practice management project.

## Results

A total of 636 patients completed the questionnaire, 383 (60%) from an obstetric practice and 253 (40%) from a pediatric practice. The majority expressed that they were not interested in Telehealth (N=407, 64%), with 228 (36%) stating interest and one missing result. Of the 221 respondents interested in Telehealth, 55.7% (N=123) indicated that they would use an iPhone, 38.9% (N=86) an Android device, 3.6% (N=8) a computer, and 1.8% (N=4) other devices. Of the 269 respondents, there was an even divide between those who would prefer audiovisual services (N=142, 52.8%) and those who would prefer audio or telephone only (N=127, 47.2%). Cronbach’s alpha for the survey questions was 0.620, and the values for individual items are listed in Table 1.

Results are presented in Table 2. Pediatric patients’ guardians expressed greater interest in Telehealth than obstetric patients (N=117, 46.2%). Only 111 (29.1%) obstetric patients were interested in completing some prenatal care visits remotely. Although there were statistical differences noted between obstetric and pediatric patients, we found that the majority in both clinics would use an iPhone or an Android device for televisits and would prefer text over email communication. Pediatric patients had a slight preference for telephone (audio only) over audiovisual visits, whereas obstetric patients preferred audiovisual combined. The highest scores (preference toward Telehealth) were noted in the domain of enjoyment, suggesting that both pediatric and obstetric patients may decline Telehealth in favor of in-person meetings simply because they like meeting with their provider.

**Table 2:** Questionnaire results. Questionnaire results are expressed as number (N) (%) for categorical results or median±standard deviation for Likert scale results. Likert scale values ranged from 0 (strongly disagree) to 4 (strongly agree). P-values are for comparisons between obstetric and pediatric visits.

	All patients		Obstetrics		Pediatrics		P-value
I am interested in Telehealth.	635	(99.8%)					
Yes	228	(35.9%)	111	(29.1%)	117	(46.2%)	<0.005
No	407	(64.1%)	271	(70.9%)	136	(53.8%)	
Device most likely to use	221	(34.7%)					
iPhone	123	(55.7%)	39	(46.4%)	84	(61.3%)	<0.005
Android	86	(38.9%)	42	(50%)	44	(32.1%)	
Computer	8	(3.6%)	3	(36%)	5	(36%)	
Other	4	(1.8%)	0	(0%)	4	(29%)	
Contact preference	259	(40.7%)					
Text	242	(93.4%)	64	(84.2%)	178	(97.3%)	<0.005
Email	17	(7%)	12	(15.8%)	5	(27%)	
Audiovisual preference	269	(42.3%)					
Video and audio	142	(52.8%)	60	(65.2%)	82	(46.3%)	0.004
Audio/telephone only	127	(47.2%)	32	(34.8%)	95	(53.7%)	
Likert scale questions							
Equipment	3.03	±1.48	2.68	±1.37	3.57	±1.49	0.118
Internet	3.14	±1.37	2.88	±1.21	3.55	±1.48	<0.005
Data charges	2.91	±1.31	2.95	±1.17	2.84	±1.50	<0.005
Timeliness	3.04	±1.19	3.05	±1.24	3.02	±1.19	0.018
Transportation	2.07	±1.29	2.13	±1.44	1.99	±1.02	<0.005
Confidence in provider	3.01	±1.24	2.85	±1.21	3.25	±1.26	0.349
Complexity of care	2.98	±1.38	3.15	±1.51	2.73	±1.67	0.003
Enjoyment	3.23	±0.84	3.10	±0.78	3.42	±0.77	0.042

We noted significantly lower mean Likert scores (favoring in-office visits) in obstetric patients compared to pediatric patients when queried about internet access and text/data charges, suggesting that these areas may be barriers to Telehealth more so in the obstetric population. Pediatric patients were also more likely to express enjoyment of in-person visits than obstetric patients. Obstetric patients had higher mean scores (favoring Telehealth) than pediatric patients when asked about the complexity of health conditions, timeliness of Telehealth visits, and transportation issues, suggesting that these are less important barriers for obstetric patients. We found similar neutral scores for both pediatric and obstetric patients regarding equipment and confidence in the provider.

Within obstetric visits, patients who were interested in Telehealth had higher mean scores (favoring Telehealth) when asked about equipment, internet access, timeliness, confidence in care, and complexity and lower mean scores in the questions about data charges and transportation (Table 3). There was no difference in mean scores about the enjoyment of meeting in person for either those interested or disinterested in Telehealth. Within pediatric visits, we found no difference based on interest in Telehealth for questions about timeliness, confidence in provider, and complexity of care. However, higher scores (favoring Telehealth) were noted in questions about equipment, internet access, and transportation (Table 3). Lower scores (toward in-person visits) were seen in regard to enjoying in-person visits among pediatric respondents, even when interested in Telehealth.

**Table 3:** Likert score for questionnaire. Survey questions in obstetric and pediatric visits, compared for responses by interest in Telehealth (yes or no). Values are mean Likert scale scores (1 toward in-person visit and 5 toward Telehealth) ± standard deviation.

	Obstetrics					Pediatrics				
	Interested		Not interested		P-value	Interested		Not interested		P-value
Equipment	4.25	±0.60	2.04	±1.03	<0.005	4.22	±1.07	3	±1.57	<0.005
Internet	4.21	±0.54	2.34	±0.97	<0.005	4.22	±1.08	2.96	±1.53	<0.005
Data charges	2.90	±1.69	2.97	±0.89	<0.005	2.89	±1.56	2.81	±1.44	0.081
Timeliness	4.32	±0.67	2.52	±1.02	<0.005	3.53	±0.98	2.58	±1.04	0.695
Transportation	1.45	±0.63	2.4	±1.58	<0.005	2.13	±1.16	1.87	±0.87	<0.005
Confidence in provider	4.27	±0.68	2.27	±0.84	<0.005	3.85	±1.06	2.76	±1.20	0.471
Complexity of care	4.57	±1.15	2.57	±1.23	<0.005	2.85	±1.72	2.61	±1.62	0.185
Enjoyment	2.95	±0.84	3.16	±0.74	0.777	3.18	±0.84	3.62	±0.65	<0.005

Mean scores for the sample and for obstetric and pediatric visits when Likert scaled items were collapsed into the three domains of cost, access, and attitude are shown in Table 4. In the entire sample, patients who were more interested in Telehealth had higher mean scores (favoring Telehealth) in the domain of cost. Of the obstetric patients, significant differences and higher scores (favoring Telehealth) were seen in patients interested in Telehealth in two domains: cost and attitude. For pediatric visits, significant differences and higher scores (favoring Telehealth) for those interested in Telehealth were only seen in the domain of attitude.

**Table 4:** Cost, access, and attitude analysis. Mean combined scores in the domains of cost, access, and attitude for all patients and obstetric and pediatric patients compared by interest in Telehealth. Values are mean±standard deviation.

	Interested		Not interested		P-value
All patients					
Cost	3.78	±0.92	2.61	±0.86	0.003
Access	2.86	±0.66	2.38	±0.64	0.396
Attitude	3.60	±0.80	2.78	±0.83	0.957
Obstetrics					
Cost	3.79	±0.79	2.45	±0.63	<0.005
Access	2.88	±0.48	2.45	±0.55	0.947
Attitude	3.93	±0.59	2.66	±0.86	<0.005
Pediatrics					
Cost	3.78	±1.03	2.92	±1.13	0.271
Access	2.83	±0.80	2.22	±0.77	0.710
Attitude	3.29	±0.85	3.00	±0.75	0.022

Logistic regression analysis for interest in Telehealth showed a significant contribution of the three domains of cost, access, and attitude (P<0.005 for each) for the entire sample. When analyzed for obstetric patients, we found similar significance across the three domains. However, when modeled for pediatric patients only, cost and access contributed to interest (P<0.005), but attitude was not significant (P=0.253).

## Discussion

At times, healthcare changes are proposed without consideration of the acceptability in the targeted population of such policies. Telehealth was rapidly advanced as a mainstream means of accessing care during the COVID-19 pandemic, with many states, including California, adopting emergency provisions to ensure payment to Telehealth providers [12]. Our study shows that despite the many advantages of Telehealth in practical terms, it is not well accepted in low socioeconomic groups in California, and the reasons go beyond the limitations to access of equipment and internet services. To improve access to healthcare, decrease costs, and improve patient-provider relationships without compromising participation and satisfaction in the lower socioeconomic and rural groups, it is imperative to understand issues of acceptability in the target population.

In the modern society of the United States, healthcare is in competition with work/personal responsibilities of patients, which leads to missed appointments [13]. Often, routine healthcare maintenance is ignored due to time away from work, which has the potential for downstream effects of diseases being diagnosed at a later time with increasing severity and complications [14]. Telehealth has the ability to bridge this gap and help stratify patients who need further evaluation from an in-office examination without wasting patients’ time and office resources [15].

Here, we surveyed a low socioeconomic demographic of California Medicaid (Medi-Cal) obstetric and pediatric practice and found that cost, access, and attitude all contribute to interest in Telehealth. Previous studies have shown that access to technology such as smartphones or internet service is a limiting factor to Telehealth services for low socioeconomic populations [16]. We found that technology was readily available for our low socioeconomic population. Our patients expressed enjoyment of in-office visits, which leads to less interest in Telehealth. Cultural beliefs and attitudes play a large perception in Western medicine for low socioeconomic groups, and beliefs may influence trust in physicians and medicine in general [17]. We cannot ignore the more primitive influences of the human psychological construct while trying to change healthcare delivery systems.

While Telehealth has the ability to reduce gaps in our current healthcare system, providers must weigh patient preferences for Telehealth and adapt to the cultural specificities of that population. Despite Telehealth being shown to have the benefits mentioned above, some patients may still not want to engage in this type of healthcare delivery for various reasons. Patients may lack confidence in themselves performing medical tasks or may just simply prefer and enjoy meeting with their providers in person. There is a movement to impart more Telehealth services, but if patient preferences go unrecognized, this movement may further compound current minority healthcare disparities.

The limitations of our study included looking at only two patient fractions in low socioeconomic medicine. We mainly focused on younger women and pediatric patients’ parents/guardians, which could have been comprised of mainly women in our healthcare population. Therefore, different preferences may exist for male and elderly populations. In addition, our patient population is largely Hispanic, with a set of cultural norms that structure which family members are present for healthcare visits and family care. Immigration status was not queried in the original questionnaire, which may also influence perceptions regarding Telehealth care.

## Conclusions

Despite readily available technology and access to Telehealth visits in low socioeconomic patients, in-person healthcare may be preferred by this patient population. In the world of changing healthcare delivery and epidemics, in-person visits are an important option for disadvantaged patients that are vital to their care. Transitioning to a Telehealth-only practice may potentially worsen already present healthcare disparities among low socioeconomic patients and challenge already present obstacles with patient compliance. Before the application of new policies, healthcare providers need to examine and understand their patient population and how new modalities will affect their healthcare.

## Appendices

Table 5 shows the patient questionnaire answered using a 5-point Likert scale from strongly disagree (1) to strongly agree (5) with survey statements.

**Table 5:** Patient questionnaire.

	Strongly disagree	Disagree	Neutral	Agree	Strongly agree
I have the equipment (smartphone or computer) needed for Telehealth.					
I have internet access that is able to support televideo visits.					
I am worried about the cost of telehealth services such as text or data charges.					
I am confident that my provider will be able to care for me through telehealth.					
My health conditions are too complicated for telehealth.					
I enjoy meeting with my provider in person.					
Telehealth will help me see my provider more timely or more often.					
I have a hard time making it to my visits due to transportation problems.					
